# Report of Health Officers of District of Columbia

**Published:** 1880-10

**Authors:** 


*Report of the Health Officers of District of Columbia.*—By Smith Townshend, M. D., 1880.

A very interesting and instructive report, exhibiting the management and the results of the Health Department of Washington City, Georgetown, &c.

